# Lysophosphatidylcholine acyltransferase 1 upregulation and concomitant phospholipid alterations in clear cell renal cell carcinoma

**DOI:** 10.1186/s13046-017-0525-1

**Published:** 2017-05-12

**Authors:** Yiqing Du, Qiang Wang, Xingzhong Zhang, Xiaofeng Wang, Caipeng Qin, Zhengzuo Sheng, Huaqi Yin, Changtao Jiang, Jing Li, Tao Xu

**Affiliations:** 1 Department of Urology, Peking University People’s Hospital, No. 11 Xi Zhi Men South Street, Beijing, 100044 China; 2 Department of Physiology and Pathophysiology, School of Basic Medical Sciences, Peking University, Key Cardiovascular Science, Ministry of Education and Beijing Key Laboratory of Cardiovascular Receptors Research, Beijing, China; 3 Department of Urology, Peking University International Hospital, Beijing, China; 4 Department of Gastroenterology, Peking University People’s Hospital, No. 11 Xi Zhi Men South Street, Beijing, 100044 China

**Keywords:** Carcinoma, Renal cell, Cancer lipidomics, LPCAT1, Phospholipid

## Abstract

**Background:**

The involvement of lipid metabolism in tumourigenesis and the progression of clear cell renal cell carcinoma (ccRCC) have been reported. However, the role of phospholipid profile alterations in ccRCC has not yet been systematically explored. In the present study, we compared the phospholipid compositions between ccRCC and paired normal renal tissues.

**Methods:**

The phospholipid compositions of paired ccRCC and normal renal tissues were evaluated using liquid chromatography tandem mass spectrometry (LC/MS/MS). To evaluate the mRNA and protein levels of lysophosphatidylcholine acyltransferase (LPCAT), which converts lysophosphatidylcholine (LPC) to phosphatidylcholine (PC), qRT-PCR, western blotting and immunohistochemistry were performed. The correlations of LPCAT1 expression with clinicopathological features and prognosis were assessed. In addition, siRNAs were used to knockdown LPCAT1 expression in ccRCC cell lines, and its effect on cell proliferation, cell cycle, migration and invasion were investigated.

**Results:**

The phospholipid compositions of ccRCC and normal renal tissues were significantly different. Multiple LPC species were decreased and corresponding PC species were increased in cancer tissues. The mRNA and protein levels of LPCAT1 were up-regulated in ccRCC tissues compared with normal renal tissues, and LPCAT1 expression was significantly correlated with unfavourable pathological features (higher tumour grade, higher TNM stage and larger tumour size) and overall survival. In cell line experiments, LPCAT1 knockdown depleted PCs, inhibited cell proliferation, migration and invasion and induced cell cycle arrest at the G0/G1 phase.

**Conclusion:**

Selective changes in PC and LPC composition were observed in ccRCC tissues. The overexpression of LPCAT1 promotes the development and progression of ccRCC, likely through the conversion of LPC to PC.

**Electronic supplementary material:**

The online version of this article (doi:10.1186/s13046-017-0525-1) contains supplementary material, which is available to authorized users.

## Background

Clear cell renal cell carcinoma (ccRCC) is the most common type of renal malignancy in adults. Due to a lack of early-warning signs, approximately 30% of ccRCC patients present with metastatic disease at diagnosis [1, 2]. Unfortunately, there are no satisfactory treatment options for patients with advanced-stage disease, as ccRCC is inherently resistant to radiotherapy and chemotherapy [2]. Thus, understanding ccRCC pathogenesis and establishing novel potential therapeutic approaches are of great clinical importance.

Apart from genetic variation, the involvement of lipid metabolism in the tumourigenesis and progression of ccRCC has recently been suggested. Ohno et al. [3] conducted a study enrolling 364 patients with ccRCC and concluded that higher preoperative levels of blood cholesterol are associated with better cancer-specific survival. The results of previous studies also indicated that serum cholesterol, high-density lipoprotein cholesterol and low-density lipoprotein cholesterol levels are correlated with the risk and pathological characteristics of RCC [4, 5]. In addition, several enzymes controlling the synthesis and metabolism of fatty acids and cholesterols are reported to play important roles in the tumourigenesis and progression of this disease [6–8].

As a main component of the cell membrane, phospholipids are essential for maintaining normal cellular structures and biological functions [9–11]. The involvement of phospholipids in the development and progression of some malignancies has been reported [12]. Several studies have investigated the alterations of phospholipids in ccRCC. Lin et al. [13] performed a LC-MS-based serum metabolomics analysis in ccRCC patients and healthy controls, and indicated that RCC is closely associated with disturbed phospholipid catabolism and sphingolipid metabolism. Cifkova et al. [14] and Saito et al. [15] investigated the phospholipid compositions in RCC and normal renal tissues, and found significantly difference among two groups. However, the mechanisms and effects of phospholipid alterations in ccRCC are not well understood.

In the present study, we performed liquid chromatography tandem mass spectrometry (LC/MS/MS) to compare the phospholipid composition of ccRCC and adjacent normal renal tissues, and conducted functional analyses in ccRCC cell lines to elucidate the biological effects of LPCAT1, which plays important role in the metabolism of phospholipid, in the development and progression of ccRCC.

## Methods

### Patients and clinical specimens

The protocol utilized in the present study was reviewed and approved by the Ethics Committee of Peking University People’s Hospital (No. 2016PHB073), and informed consent was obtained from all participants. Thirty patients who underwent partial or radical nephrectomy during February 2016 to May 2016 were enrolled in the present study. The mean age of the patients was 59.4 ± 11.5 years (ranging from 37 to 81 years), and 70.0% of the subjects were males. All participants were pathologically diagnosed with ccRCC, and none of the patients received antitumour therapy prior to surgery. Paired ccRCC and normal renal tissues were snap frozen in liquid nitrogen immediately after resection and stored at -80 °C. Subsequently, the resected specimens were used for lipidomics analysis and RNA or protein isolation.

### Analysis of tissue and cell lipidomics by LC/MS/MS

The LC/MS/MS system used in the present study included an Ultimate 3000 ultra-high-performance liquid chromatograph and a hybrid quadrupole orbitrap mass spectrometer Q Exactive (Thermo Fisher Scientific, Waltham, MA). Full scan phospholipid quantification was performed, and the data were normalized based on the tissue weight or cell number. The normalized data were further exported to MetaboAnalyst 3.0 for multivariate analysis. Both principal component analysis (PCA) and partial least-squares-discriminant analysis (PLS-DA) were used for modelling the difference between tumour and normal tissues. Details are provided in the Additional file 1.

### RNA extraction and quantitative real-time PCR (qRT-PCR)

Total RNA from tissue and cell samples was extracted using TRIzol Reagent (Invitrogen, Carlsbad, CA, USA). Complementary DNA was generated from 1.5 μg of total RNA using SuperScript III (Invitrogen, Carlsbad, CA, USA) according to the manufacturer’s instructions. Subsequently, qRT-PCR was performed on an Opticon 2 PCR instrument (Bio-Rad Laboratories, Hercules, CA, USA) using Brilliant II SYBR® Green QPCR Master Mix (Agilent Technologies, Santa Clara, CA, USA). The transcription levels of target genes were normalized to GAPDH and calculated using the 2^-△△Ct^ method. The experiments were repeated at least three times. The primer sequences used in the present study were summarized in Table 1.

**Table 1:** RT-PCR primers

Genes		Primer sequence
LPCAT1	5’	CACAACCAAGTGGAAATCGAG
	3’	GCACGTTGCTGGCATACA
LPCAT2	5’	AAAGCGAACAACATCAGGAG
	3’	GAGCTGGCAGAAAGTAAGCA
LPCAT3	5’	ATCACTGCCGTCCTCACTAC
	3’	AGTCAACAGCCAAACCAATC
LPCAT4	5’	TTGTGGATGTGGAGTTCCTT
	3’	ACTTTTCCCAGTTCCCAGAG
GAPDH	5’	GGGCTGCTTTTAACTCTGGT
	3’	TGATTTTGGAGGGATCTCGC

### Protein extraction and western blotting

Total protein from tissue and cell samples was lysed in RIPA buffer (Solarbio, Shanghai, China) containing freshly added protease and phosphatase inhibitor cocktail (Thermo Fisher Scientific, Waltham, MA), and the protein concentration was quantified using the BCA Protein Assay Kit (Solarbio, Shanghai, China). Subsequently, equal quantities of protein were separated using sodium dodecyl sulphate polyacrylamide gel electrophoresis (SDS-PAGE) and transferred onto nitrocellulose membranes. After blotting with 5% non-fat milk or BSA, the membranes were incubated with anti-LPCAT1 (1:1000; Proteintech, Chicago, IL, USA) or anti-GAPDH (1:3000; Cell Signalling Technology, Danvers, MA, USA) overnight at 4 °C, followed by incubation with horseradish peroxidase-conjugated secondary antibodies for 1 h at room temperature. The immunoreactive bands were visualized using a chemiluminescence kit according to the manufacturer’s instructions (ECL; Millipore, Billerica, MA, USA). The signal intensities were quantitated using Quantity One software (Bio-Rad Laboratories, Hercules, CA, USA), and GAPDH was used as an internal control. The experiments were repeated at least three times.

### Human ccRCC tissue array and immunohistochemistry (IHC)

A ccRCC tissue microarray (TMA) was purchased from Shanghai Outdo Biotech (Shanghai, China) to detect the expression of LPCAT1. This TMA contained 180 spots from 150 ccRCC patients, including paired ccRCC tissues and corresponding normal renal tissues from 30 patients and 120 ccRCC tissues from 120 patients. The demographic and clinicopathological data for each patient were available. The follow-up time ranged from 5.4 to 7.5 years, and all patients were followed up until August 2015. IHC was performed as previously described [16], and the rabbit polyclonal LPCAT1 antibody (Proteintech, Chicago, IL, USA) was used at a dilution of 1:600. The colorectal adenocarcinomas specimens were used as positive control. The intensity was quantified by two pathologists blinded to the clinical status of the patients, and the results were categorized according to the staining intensity of LPCAT1 as follows: -, negative; +, weak; ++, moderate; and +++, intense.

### Cell culture and siRNA transfection

ACHN and 769P cell lines were purchased from the Cell Bank of the Chinese Academy of Sciences, Beijing, China. The cells were routinely cultured in MEM/EBSS or RPMI 1640 media supplemented with 10% fetal bovine serum, 100 U of penicillin and 100 μg/mL of streptomycin at 37 °C in a humidified 5% CO_2_ atmosphere. To knock down LPCAT1 expression, ACHN and 769P cells were transfected with siRNAs using Lipofectamine® 3000 (Invitrogen, Carlsbad, CA, USA) according to the manufacturer’s instructions. The following sequences were used for the two LPCAT1 siRNAs and the non-specific control siRNA (NC): si-LPCAT1-1: 5’-GAUCCAGUAUAUACGGCCUTT-3’ (sense), 5’-AGGCCGUAUAUACUGGAUCTT-3’ (antisense); si-LPCAT1-2: 5’-CCUGCCUAAUUACCUUCAATT-3’ (sense), 5’-UUGAAGGUAAUUAGGCAGGTT-3’ (antisense); and si-NC: 5’-UUCUCCGAACGUGUCACGUTT-3’ (sense), 5’-ACGUGACACGUUCGGAGAATT-3’ (antisense).

### Cell proliferation assay

Cell proliferation was analysed using a Cell Counting Kit-8 (CCK-8, Dojindo Laboratories, Kumamoto, Japan) and a colony formation assay. For the CCK-8 assay, the cells were seeded onto 96-well plates at a density of 2000 cells per well and subsequently transfected with the corresponding siRNAs. At the indicated time points, 10 μL of CCK-8 was added to each well and incubated at 37 °C for 2 h. Subsequently, the absorbance of live cells was measured at 450 nm using a microplate reader (Bio-Rad Laboratories Inc., Tokyo, Japan). For the colony formation assay, the cells were seeded onto 6-well plates at a density of 2000 cells per well and transfected with the indicated siRNAs. The transfected cells were cultured for 2 weeks, and subsequently, the cells were fixed with 4% paraformaldehyde and stained with crystal violet. The experiments were repeated at least three times.

### Cell cycle assay

The cell cycle assay was performed using a cell cycle staining kit (Multi Sciences, Hangzhou, China) according to the manufacturer’s instructions. Briefly, the cells were harvested and washed with PBS. After incubating with DNA staining solution and permeabilization solution for 30 min at room temperature, the cells were measured using a CytoFLEX flow cytometer (Beckman Coulter, Miami, FL, US). The cell cycle distribution was analysed using ModFit software (Verity Software House, Topsham, ME). The experiments were repeated at least three times.

### Cell migration and invasion assay

The cell migration and invasion assays were performed using cell culture inserts and a Matrigel Invasion Chamber (8 μm pore size; Corning). A total of 5 × 10^4^ cells were resuspended in 200 μL of serum-free medium and seeded into the upper chamber; 600 μL of medium containing 10% FBS was filled in the bottom chamber. The transwell plates were cultured at 37 °C for 20 h (migration) or 36 h (invasion), and the cells on the upper surface of the membrane were removed. The cells that passed through the membrane were fixed with 4% paraformaldehyde and stained with crystal violet. The migrating or invading cells were counted under a microscope in five random fields. All assays were repeated at least three times.

### Statistical analysis

Continuous variables are reported as the means ± standard error of the mean (SEM), and the categorical variables are presented as proportions. Student’s *t*-test or *χ* ^2^ test was used to compare the differences between different experimental groups. The association between LPCAT1 expression and the clinicopathological characteristics was tested using the Kruskal-Wallis test or the Cochran-Mantel-Haenszel test, as appropriate. The Kaplan-Meier method and the log-rank test were performed to detect the survival differences between the groups. All statistical analyses were performed using SPSS software, version 13.0 (SPSS Inc., Chicago, IL, USA). All p values were 2-tailed, and *p* < 0.05 was considered statistically significant.

## Results

### The phospholipid compositions in ccRCC are different from those in the corresponding normal renal tissues

**Fig. 1:**
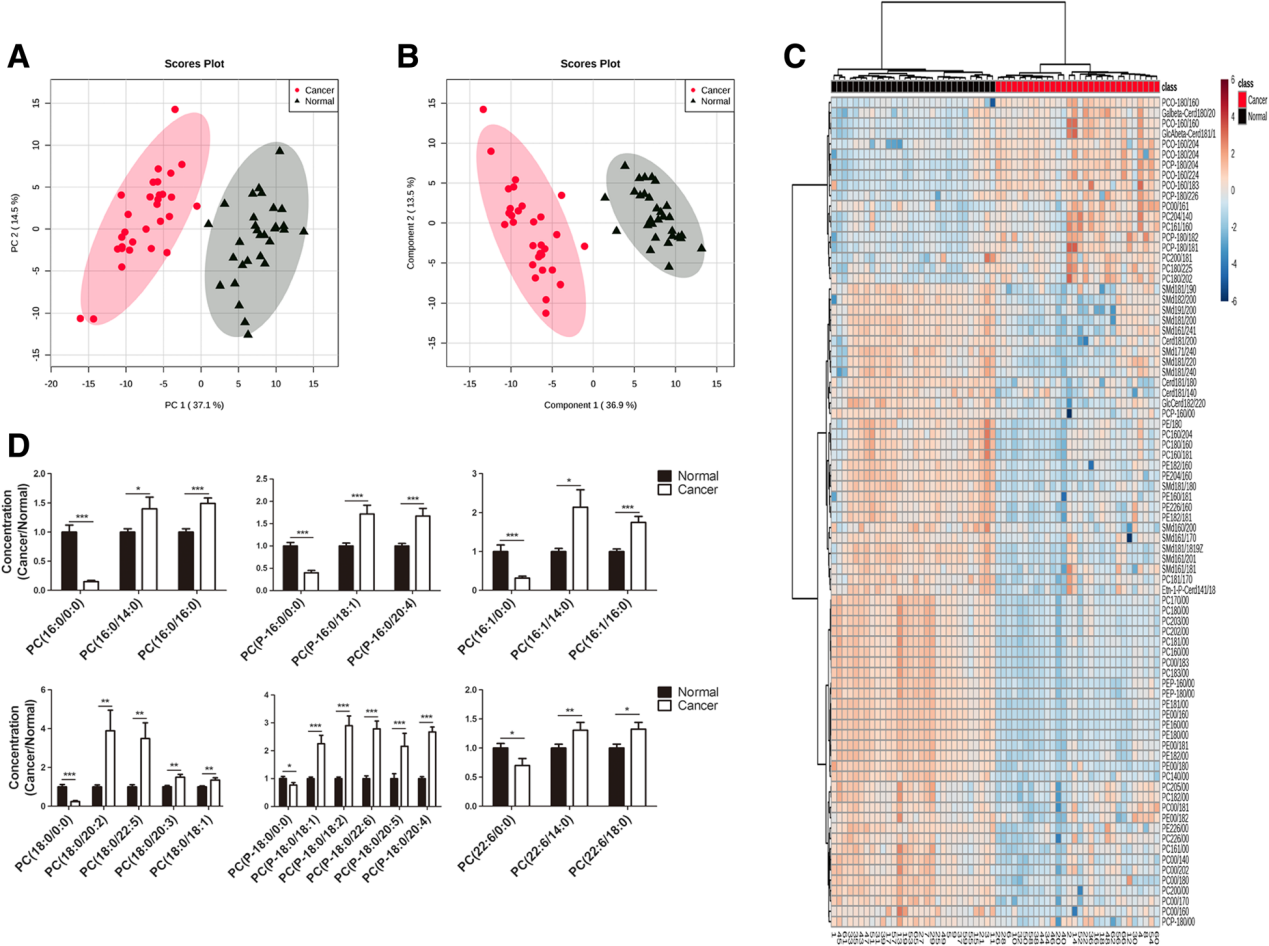
The phospholipid compositions in ccRCC are different from those in the corresponding normal tissues. **a** PCA and (**b**) PLS-DA score plots based on the phospholipid profile. (*red circle* clear cell RCC tissues, ▲ normal renal tissues). **c** Clustering results shown as a heatmap (hierarchical clustering performed using MetaboAnalyst 3.0). The colour of each section is proportional to the significance of the change in metabolites (*red*, up-regulated; *blue*, down-regulated). **d** Significantly altered LPC and corresponding PC species in 30 paired ccRCC tissues compared with normal renal tissues. PC (O-n_1_:m_1_/n_2_:m_2_) stands for ether type PC, PC (P- n_1_:m_1_/n_2_:m_2_) stands for plasmalogen type PC. The values are presented as the means ± SEM. *, *P* < 0.05; **, *P* < 0.01; ***, *P* < 0.001

We collected paired ccRCC and normal renal tissues from 30 patients with ccRCC and evaluated the phospholipid compositions using LC/MS/MS. A total of 160 phospholipid species were detected, including 85 PC, 24 phosphatidylethanolamine (PE), 23 sphingomyelin (SM) and 28 ceramide molecules. First, multivariate statistical analyses were performed to identify the difference between ccRCC and normal tissues. Both unsupervised PCA and supervised PLS-DA could discriminate ccRCC tissues from matched normal tissues based on the phospholipid profile (Fig. 1a, b). As shown in Fig. 1c, the cancerous tissues showed different phospholipid compositions compared to normal tissues. Multiple LPC species were decreased in cancerous tissues, and an increase of corresponding PC species (containing same fatty acid chains with the LPC) was observed in the cancerous tissues (Fig. 1d).

### LPCAT1 is up-regulated in ccRCC tissues

**Fig. 2:**
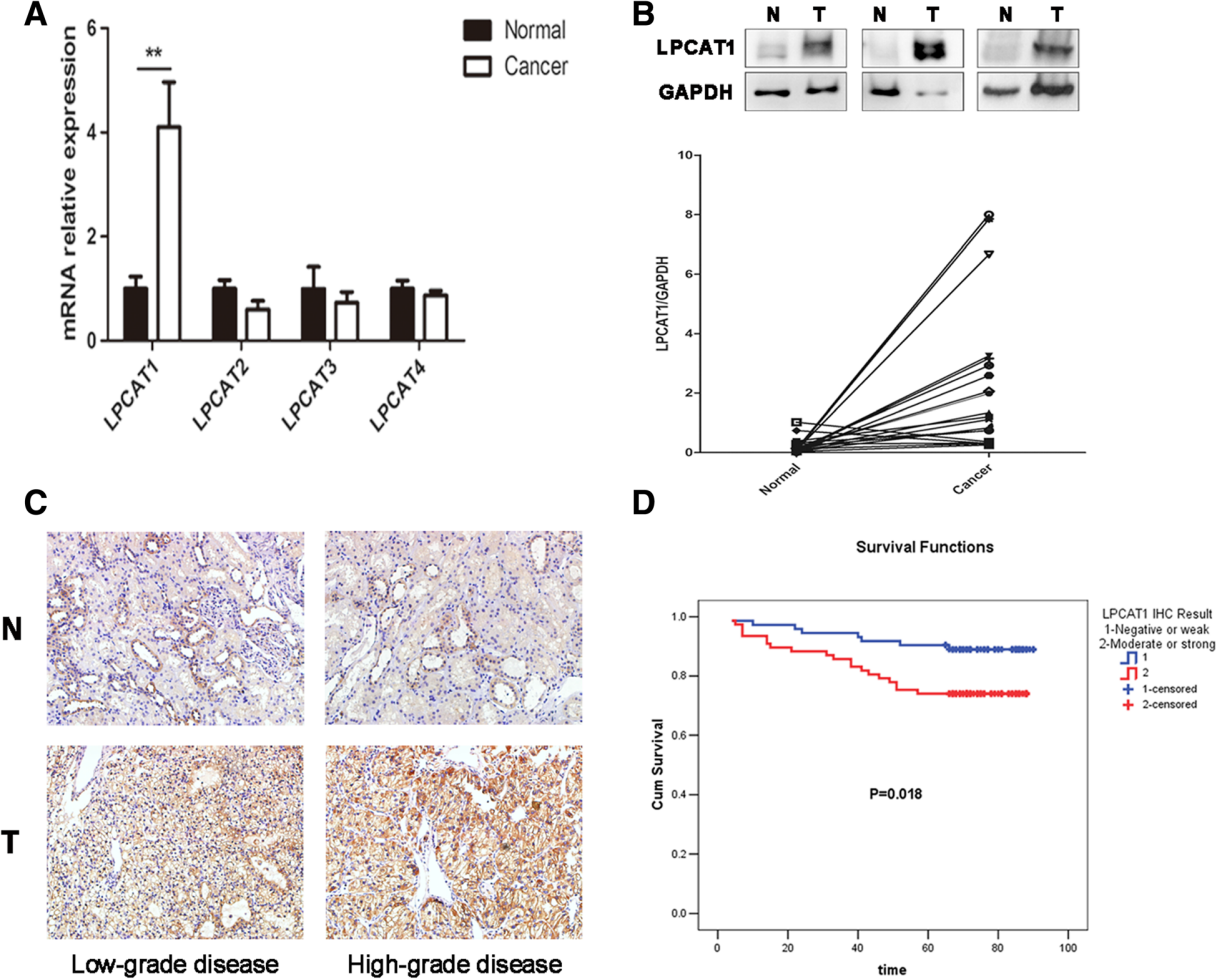
LPCAT1 is up-regulated in ccRCC tissues and correlates with the overall survival of patients. **a** qPCR analyses of LPCAT1-4 in 20 paired ccRCC and normal renal tissues. **b** Western blot analysis of LPCAT1 in 20 paired ccRCC and normal renal tissues. The *upper panel* shows the results of three representative paired tissues. N and T represent normal renal and ccRCC tissues, respectively. The *lower panel* shows the results in 20 paired tissues. GAPDH was used as an internal standard. **c** Representative IHC results of LPCAT1 expression in low-grade ccRCC tissues, high-grade ccRCC tissues and paired normal renal tissues. Positive LPCAT1 staining were detected in the membrane and cytoplasm of cancerous tissues, while negative LPCAT1 staining were detected in the normal renal tissues. N and T represent normal renal and ccRCC tissues, respectively. **d** Patients in comparative LPCAT1-high groups had lower overall survival times than those in comparative LPCAT1-low groups (*p* = 0.018)

Based on the results of lipidomics, we hypothesized that the phospholipid composition alterations in ccRCC tissues reflect increased PC formation from LPC. Thus, the expression levels of LPCATs, which catalyse the conversion from LPC to PC, were analysed in patient tissues. First, we examined the mRNA expression of four LPCATs in 20 paired ccRCC and normal renal tissues. The qRT-PCR results showed that LPCAT1 was significantly up-regulated in ccRCC tissues, while the expression of LPCAT2, LPCAT3 and LPCAT4 was comparable between the two groups (Fig. 2a). We further compared the LPCAT1 protein expression in the same cohort and observed consistent results (Fig. 2b). Subsequently, IHC assays were performed on the ccRCC TMA. Figure 2c shows representative images of LPCAT1 expression in ccRCC and matched normal renal tissues. Morphologically, strong or moderate LPCAT1 signals were detected in most of cancerous tissues, while negative or weak signals were present in the normal tissues. Among the 150 ccRCC tissue spots included in the TMA, 147 (98.00%) spots were positive for LPCAT1 (staining in the membrane and cytoplasm of cancer cells), while only 4/30 (13.33%) normal renal tissue spots showed positive signals (staining in the renal tubular) (*χ* ^2^ test, *p* < 0.001). Taken together, the expression of LPCAT1 is up-regulated in ccRCC tissues compared with that in matched normal renal tissues, which may at least be partly responsible for the phospholipid profile alterations observed in ccRCC.

### LPCAT1 expression correlates with the clinicopathological features and overall survival in ccRCC patients

Because LCPAT1 is up-regulated in ccRCC tissues, this protein might play a role in disease progression. To test this idea, the correlation between multiple clinicopathological features and LPCAT1 expression was investigated based on the IHC results of the ccRCC TMA. Tumour size, stage and grade were evaluated according to the 2010 American Joint Committee on Cancer guidelines TNM classification [17] and the International Society of Urological Pathology grading system [18, 19], respectively. Because the number of ccRCC spots with negative LPCAT1 IHC staining was limited, we divided the cohort of 150 ccRCC cases into three groups based on LPCAT1 IHC intensity: negative/weak (-/+), moderate (++) and intense (+++). As shown in Table 2, statistical analyses indicated that the expression of LPCAT1 was positively correlated with tumour grade (*p* < 0.0001), stage (*p* = 0.046) and size (*p* = 0.003), and there was no association between LPCAT1 expression and age or gender. In addition, the relationship between LPCAT1 expression and the overall survival of ccRCC patients was evaluated. A total of 150 patients were classified into two groups according to the LPCAT1 IHC intensity: low-LPCAT1 (-/+) and high-LPCAT1 (++/+++). Kaplan-Meier survival curves showed that the high expression of LPCAT1 in tumours correlated with worse survival in ccRCC patients (log-rank test, *p* = 0.018) (Fig. 2d). These findings indicated that the overexpression of LPCAT1 might contribute to ccRCC progression.

**Table 2:** Correlation between clinicopathological features and LPCAT1 expression in ccRCC patients

Clinicopathological parameter	Result of LPCAT1 IHC			*P* Value
	-/+	++	+++	
Age (year)				
< 60	47 (54.02%)	35 (40.23%)	5 (5.75%)	0.122^b^
60 ≤ age ≤ 70	15 (35.71%)	18 (42.86%)	9 (21.43%)	
> 70	11 (52.38%)	7 (33.33%)	3 (14.29%)	
Gender				0.860^a^
Male	52 (48.60%)	42 (39.25%)	13 (12.15%)	
Female	21 (48.84%)	18 (41.86%)	4 (9.30%)	
Grade				<.0001^b^
1	48 (77.42%)	12 (19.35%)	2 (3.23%)	
2	22 (31.88%)	42 (60.87%)	5 (7.25%)	
3–4	3 (15.79%)	6 (31.58%)	10 (52.63%)	
Stage				0.046^b^
1	65 (53.28%)	46 (37.70%)	11 (9.02%)	
2	3 (18.75%)	9 (56.25%)	4 (25.00%)	
3–4	5 (41.67%)	5 (41.67%)	2 (16.66%)	
Diameter				0.003^b^
≤ 4	41 (54.67%)	29 (38.67%)	5 (6.66%)	
4 < Diameter ≤ 7	28 (52.83%)	19 (35.85%)	6 (11.32%)	
Diameter > 7	4 (18.18%)	12 (54.55%)	6 (27.27%)	

^a^Kruskal-Wallisl Test

^b^Cochran-Mantel-Haenszel Test

### Knockdown of LPCAT1 changes the PC composition of ccRCC cells

To investigate the function of LPCAT1 in ccRCC, we used specific siRNAs to knockdown the expression of LPCAT1 in the ccRCC cell lines 769P and ACHN. Two specific siRNAs (si-LPCAT1-1 and si-LPCAT1-2) and one non-specific control siRNA (si-NC) were used in the present study. At 24 h after transfection, qRT-PCR was performed to evaluate the expression level of LPCAT1. Compared to si-NC, both specific siRNAs (si-LPCAT1-1 and si-LPCAT1-2) substantially inhibited LPCAT1 mRNA expression in ACHN cells (Additional file 2: Figure S1a). At 48 h after transfection, western blot analysis was performed, confirming that the expression level of LPCAT1 protein was significantly reduced in ACHN cells transfected with specific siRNAs (Additional file 2: Figure S1b). Similar results were also observed in 769P cells (Additional file 2: Figure S1c, d).

**Fig. 3:**
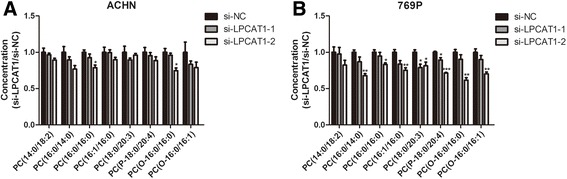
LPCAT1 knockdown changes PC composition. Altered PC species in ACHN (**a**) and 769P (**b**) cells. PC (O-n_1_:m_1_/n_2_:m_2_) stands for ether type PC, PC (P- n_1_:m_1_/n_2_:m_2_) stands for plasmalogen type PC. The results are expressed as the means ± SEM of three independent experiments. *, *P* < 0.05; **, *P* < 0.01; ***, *P* < 0.001

At 48 h after transfection, we examined the PC compositions of ccRCC cells, and confirmed the reductions of some PCs after LPCAT1 knockdown in ACHN and 769P (Fig. 3a, b). Compared with si-NC, cells transfected with specific siRNAs contained lower levels of PC(14:0/18:2), PC(16:0/14:0), PC(16:0/16:0), PC(16:1/16:0), PC(18:0/20:3), PC(P-18:0/20:4), PC(O-16:0/16:0) and PC(O-16:0/16:1).

### Knockdown of LPCAT1 suppresses the proliferation of ccRCC cells

**Fig. 4:**
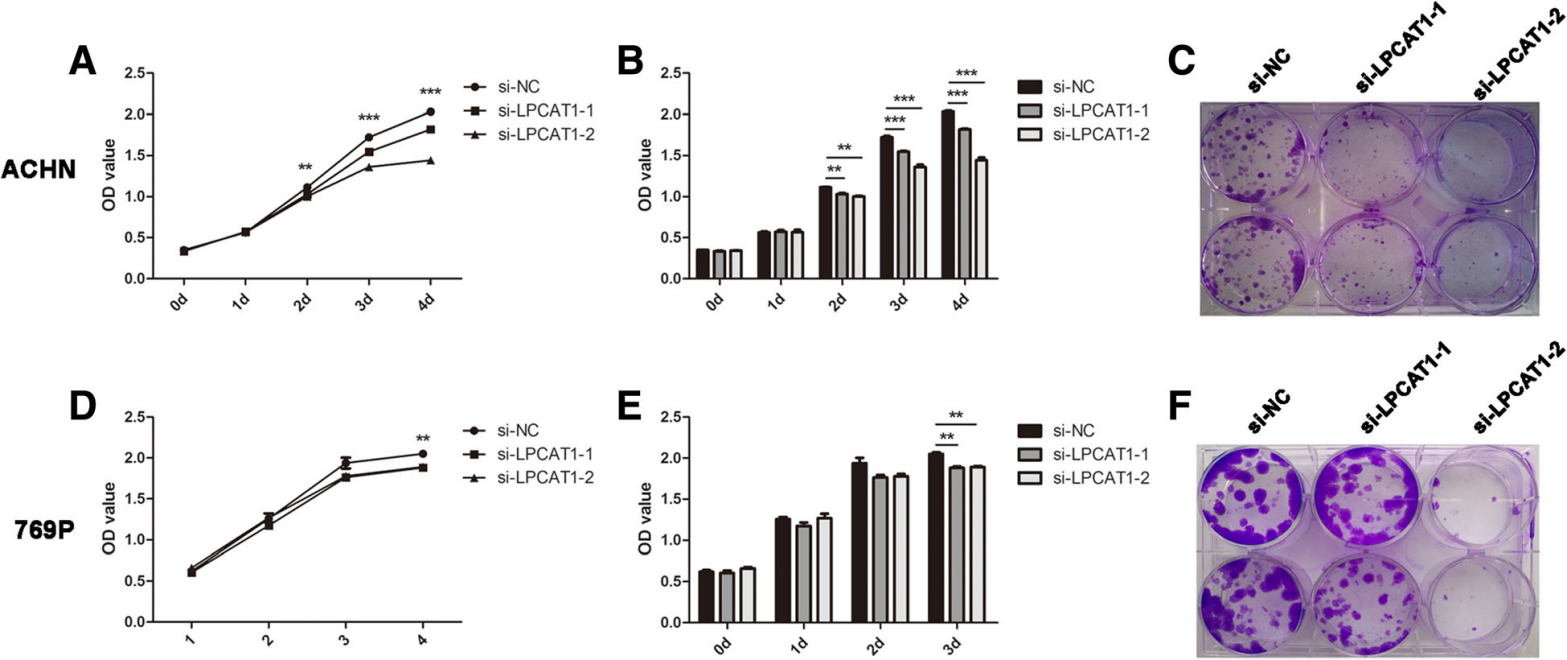
The knockdown of LPCAT1 suppresses cell proliferation. **a** and **b** CCK8 assay of LPCAT1 knockdown and control ACHN cells at the indicated times. **c** Plate colony formation assay of LPCAT1 knockdown and control ACHN cells for 2 weeks. **d** and **e** CCK8 assay of LPCAT1 knockdown and control 769P cells at the indicated times. **f** Plate colony formation assay of LPCAT1 knockdown and control 769P cells for 2 weeks. The results are expressed as the means ± SEM of three independent experiments. *, *P* < 0.05; **, *P* < 0.01; ***, *P* < 0.001

We investigated the effect of LPCAT1 on cellular proliferation using CCK8 and colony formation assays. For the CCK8 assay, ACHN and 769P cells were transfected with siRNAs, and subsequently the cell viability was monitored for 4 days. The results showed that the viability of cells transfected with si-LPCAT1-1 and si-LPCAT1-2 was significantly inhibited compared with cells transfected with si-NC (Fig. 4a, b, c, d). For the colony formation assay, cell proliferation was evaluated at 2 weeks after transfection. As shown in Fig. 4e and f, the number and size of the colonies were also significantly decreased in cells transfected with si-LPCAT1-1 and si-LPCAT1-2. Taken together, these results indicated that the knockdown of LPCAT1 suppresses the proliferation of ACHN and 769P ccRCC cells.

### Knockdown of LPCAT1 induces cell cycle arrest at the G0/G1 phase in ccRCC cells

**Fig. 5:**
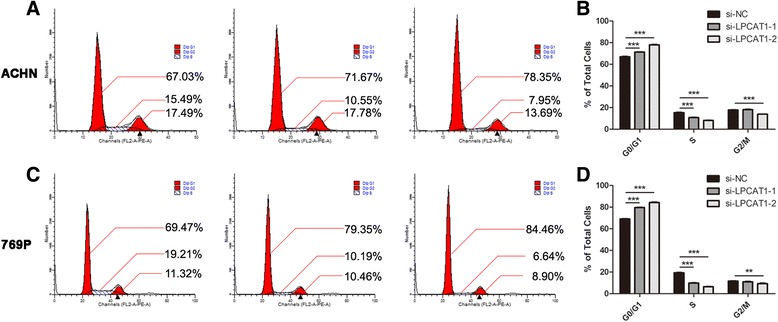
The knockdown of LPCAT1 induces cell cycle arrest at the G0/G1 phase. Flow cytometry analysis of the cell cycle in LPCAT1 knockdown and control ACHN cells. Representative histograms (**a**) and the percentage of cells at different phases (**b**). Flow cytometry analysis of the cell cycle in LPCAT1 knockdown and control 769P cells. Representative histograms (**c**) and the percentage of cells at different phases (**d**). The results are expressed as the means ± SEM of three independent experiments. *, *P* < 0.05; **, *P* < 0.01; ***, *P* < 0.001

Because the cell cycle profile can reflect cell proliferation, we evaluated whether LPCAT1 affected the cell cycle distribution of ACHN and 769P. The cells were harvested at 48 h after transfection. After incubation with the indicated staining solution, the cells were analysed using flow cytometry. As shown in Fig. 5, the knockdown of LPCAT1 led to a significant increase in the proportion of cells at the G0/G1 phase and a decrease in the proportion of cells at the S and G2/M phases. These results indicated that the knockdown of LPCAT1 could induce cell cycle arrest at the G0/G1 phase in the ACHN and 769P ccRCC cells.

### Knockdown of LPCAT1 inhibits the migration and invasion of ccRCC cells

**Fig. 6:**
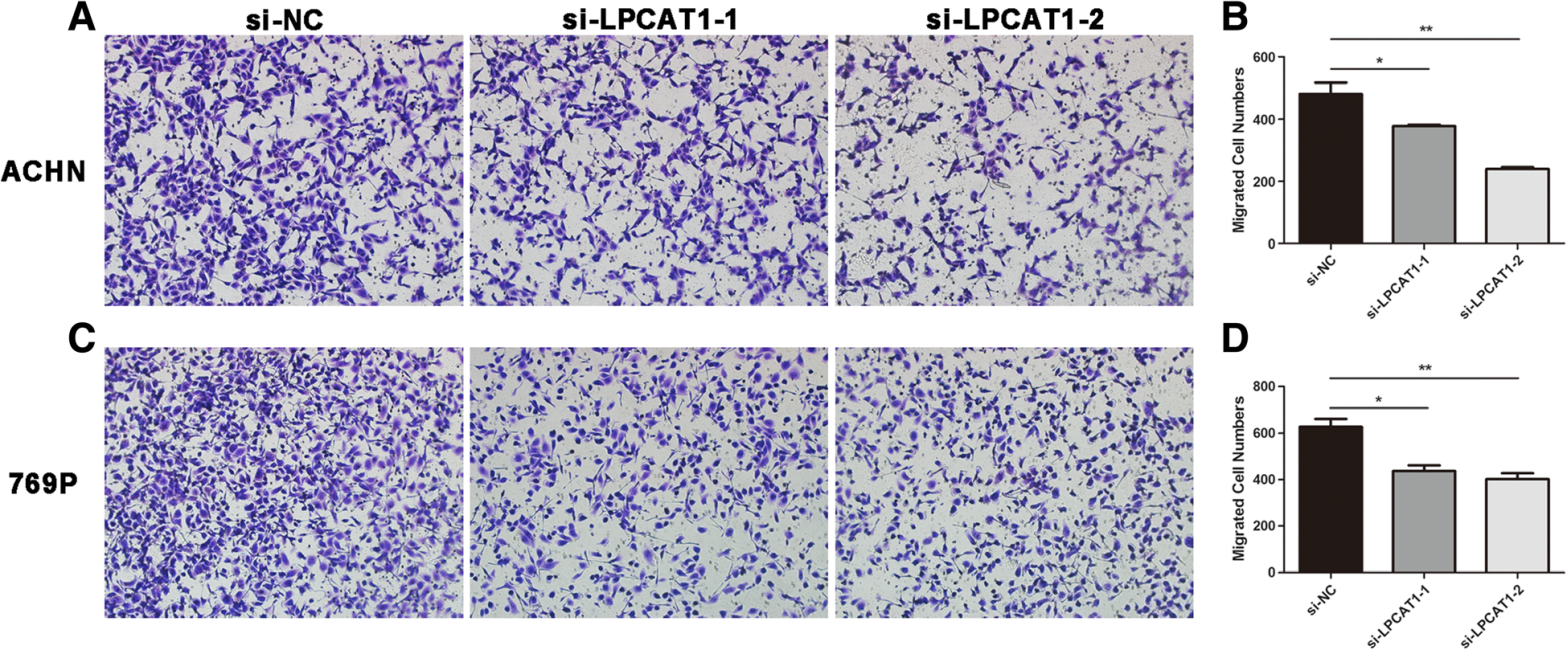
The knockdown of LPCAT1 inhibits cell migration. **a** Representative photographs of migratory ACHN cells on the membrane in the cell migration assay (magnification, ×100). **b** Average migratory ACHN cells from five random fields. **c** Representative photographs of migratory 769P cells on the membrane in the cell migration assay (magnification, ×100). **d** verage migratory 769P cells from five random fields. The results are expressed as the means ± SEM of three independent experiments. *, *P* < 0.05; **, *P* < 0.01; ***, *P* < 0.001

**Fig. 7:**
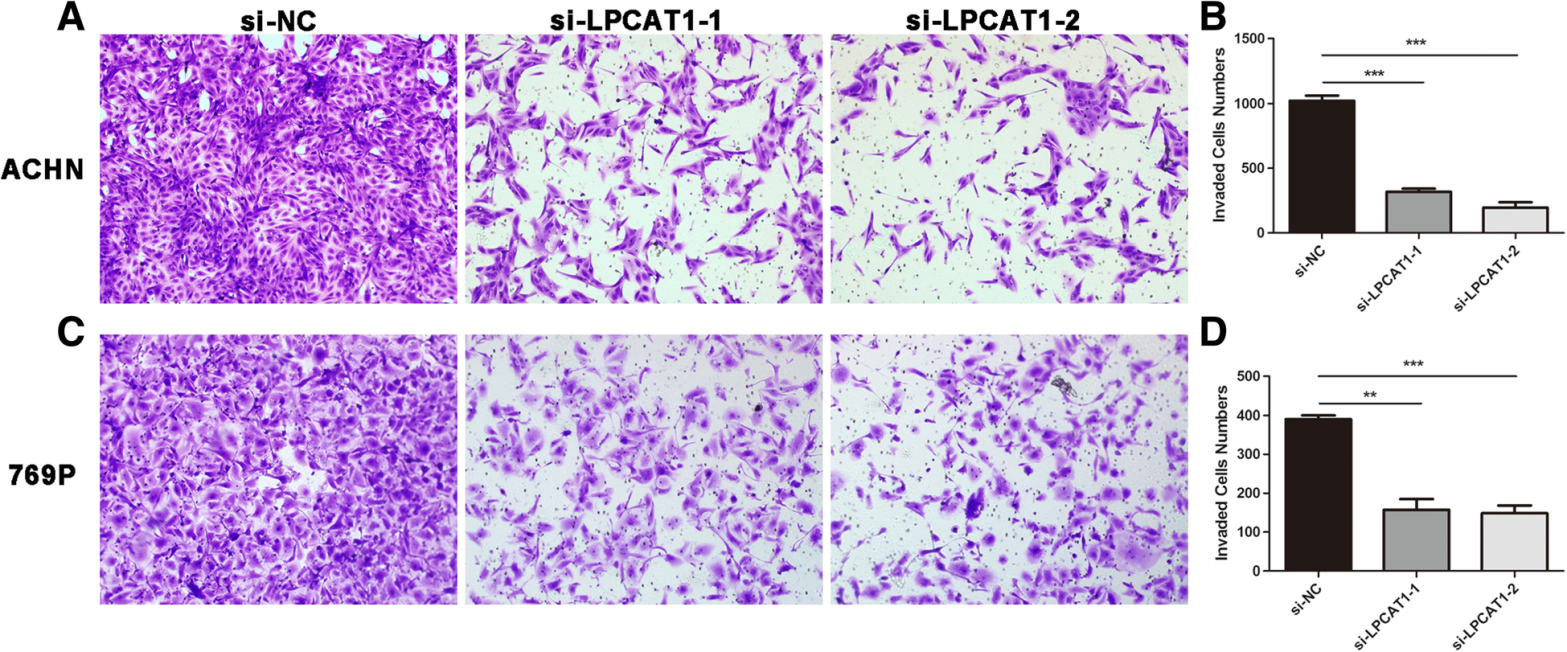
The knockdown of LPCAT1 inhibits cell invasion. **a** Representative photographs of invasive ACHN cells on the membrane in the cell invasion assay (magnification, ×100). **b** Average invasive ACHN cells from five random fields. **c** Representative photographs of invasive 769P cells on the membrane in the cell invasion assay (magnification, ×100). **d** Average invasive 769P cells from five random fields. The results are expressed as the means ± SEM of three independent experiments. *, *P* < 0.05; **, *P* < 0.01; ***, *P* < 0.001

Migration and invasion play important roles in the progression of cancers, particularly in metastasis. Thus, transwell assays were performed to evaluate the effect of LPCAT1 on cell migration and invasion. The migration assay revealed that the knockdown of LPCAT1 could inhibit the motility of ccRCC cells. Compared with the si-NC-transfected control group, the numbers of penetrating cells were reduced 21.56 and 50.20% in ACHN cells transfected with si-LPCAT1-1 and si-LPCAT1-2, respectively (Fig. 6a, b). Similar results were observed in 769P cells (Fig. 6c, d). The invasion assay showed the inhibition of cell invasion after LPCAT1 knockdown. As shown in Fig. 7a and b, the quantification analysis revealed that only 31.15 and 19.10% of ACHN cells transfected with si-LPCAT1-1 and si-LPCAT1-2 penetrated the membrane compared with ACHN cells transfected with si-NC (Fig. 7a, b). Consistent results were also observed in 769P cells (Fig. 7c, d). Overall, these results indicated that the knockdown of LPCAT1 could inhibit the migration and invasion of ACHN and 769P ccRCC cells.

## Discussion

As a relatively new concept in oncogenesis, lipid profile alterations have been implicated in the development and progression of various cancers, such as breast cancer, hepatocellular carcinoma and prostate cancer [20–23]. ccRCC is characterized by sterol storage in the tumour cytoplasm, suggesting that alterations in lipid metabolism may also be involved in the formation and progression of this disease. Phospholipid is a main component of the cell membrane, and several studies have reported the alterations of phospholipids in ccRCC. Lin et al. [13] performed a LC-MS-based serum metabolomics analysis to discriminate RCC patients from healthy controls, achieving 100% sensitivity in detection, and indicated that RCC is closely associated with disturbed phospholipid catabolism and sphingolipid metabolism. Cifkova et al [14] used hydrophilic interaction liquid chromatography coupled to electrospray ionization mass spectrometry to analysis the differences among lipid species in RCC and surrounding normal tissues from 20 kidney cancer patients. They found the lipid class concentrations were significantly different between the two groups, with PE, LPC and SM showing the highest variance. Levels of PE, SM and LPC were decreased in ccRCC tissues compared to surrounding normal tissues. Saito et al [15] compared levels of 326 lipids (including phospholipids, sphingolipids, neutral lipids and eicosanoids) in ccRCC and surrounding normal renal tissues, and found the cancerous tissues contain higher levels of ether-type PCs, cholesterol esters and triacylglycerols, and lower levels of PE, phosphatidylinositol (PI) and SM. The total levels of the PC lipids were comparable between the two groups, but 48% of the PC molecules were present in significantly higher levels in cancerous tissues. In addition, they suggested that the PE synthesis pathway is suppressed in ccRCC since several enzymes involved in this process were reduced in cancerous tissues. However, the mechanisms and effects of phospholipid alterations, especially PC species, in ccRCC are not well understood.

In the present study, we investigated the phospholipid compositions of cancer and adjacent normal renal tissues from 30 ccRCC patients and revealed obvious phospholipid alterations in cancer tissues compared with normal tissues. We found levels of PE and SM were significantly reduced in cancerous tissues, which were in line with previous studies [14, 15]. Besides, we noticed multiple LPC species decreased and corresponding PC species increased in cancerous tissues. These findings were consistent with the serum phospholipid alterations in RCC patients reported previously, which found a distinct decline of LPC in ccRCC patients [13, 24]. Therefore, we propose selective changes in PC and LPC compositions might fuel the tumourigenesis of ccRCC.

LPC and PC can convert to each other through consecutive deacylation and reacylation reactions, referred to as the Lands cycle. The crucially important enzymes controlling these biochemical processes are LPCATs and PLA_2_. LPCATs are responsible for the conversion of LPC to PC, and PLA_2_ catalyses the generation of LPC from PC [25]. To date, 4 LPCAT subtypes have been identified [26]. Among these subtypes, LPCAT1 has attracted much attention from oncology researchers. Several studies have reported that LPCAT1 is overexpressed in several kind of cancer tissues and contributes to the development and progression of cancers. Thus, we hypothesized that the accumulation of PC and reduction of LPC in ccRCC tissues likely reflects the overexpression of LPCATs, and this accumulation might promote the tumourigenesis and progression of ccRCC.

To confirm this hypothesis, we evaluated the expression of LPCATs in ccRCC tissues and matched normal renal tissues. The results showed that LPCAT1 is up-regulated at both transcript and protein levels in ccRCC tissues, while the LPCAT2, LPCAT3 and LPCAT4 levels were comparable between the two groups. Similarly, higher LPCAT1 expression was reported in several other malignant tumours compared to normal tissues, including colorectal adenocarcinoma [12], prostate cancer [23, 27, 28], hepatocellular carcinoma [22], gastric cancer [29], breast cancer [30] and oral squamous cell carcinoma [31].

In addition, we evaluated the association of LPCAT1 expression and clinicopathological characteristics and clinical outcomes. The results showed LPCAT1 expression in ccRCC was significantly correlated with unfavourable pathological features (higher tumour grade, higher TNM stage and larger tumour size) and clinical outcomes. In accordance with our findings, Zhou et al. [28] indicated the expression of LPCAT1 in metastatic prostate cancer was higher than primary prostate cancer, and the LPCAT1 level was correlated to the tumour grade and stage.

Next, we performed functional analyses in ccRCC cell lines to further evaluate the effect of LPCAT1. The in vitro knockdown experiment revealed that LPCAT1 plays a crucial role in the development and progression of ccRCC. The down-regulation of LPCAT1 could not only suppress cellular proliferation and induce cell cycle arrest but also inhibit the migration and invasion of ccRCC cells.

Taken together, these results indicated that LPCAT1 overexpression contributes to the development and progression of ccRCC. However, the underlying mechanisms remain unclear. Previous studies have indicated that the overexpression of LPCAT1 facilitates the conversion of LPC to PC, and the increased synthesis of membrane PC is required in tumourigenesis. Alterations of membrane PC levels can influence cell proliferation and membrane fluidity, which facilitate cancer cell growth and metastases [32, 33]. In the present study, we observed significant phospholipid alterations and the accumulation of several PC species in ccRCC tissues, and knockdown of LPCAT1 could deplete PCs and inhibited proliferation, migration and invasion abilities of ccRCC cell lines. Thus, the overexpression of LPCAT1 in ccRCC breaks the balance of phospholipid metabolism. Alterations of phospholipids enhance cell proliferation and membrane fluidity and eventually lead to the development and progression of ccRCC.

In conclusion, we elucidated LPCAT1 exerts an important role in the development and progression of ccRCC, likely via alterations in the phospholipid profile. To our knowledge, this study is the first to analyse LPCAT1 expression in ccRCC tissues and examine its impact on the development and progression of this disease. These findings will provide a foundation for potential novel therapeutic approaches and highlight the important role of phospholipid metabolism in ccRCC biology.

## Conclusion

Selective changes in the PC and LPC composition were observed in ccRCC tissues. The overexpression of LPCAT1 promotes the development and progression of ccRCC, likely through the conversion of LPC to PC. LPCAT1 could be a potential target molecule to inhibit the tumourigenesis and progression of ccRCC.

## Additional files

Additional file 1: Analysis of tissue and cell lipidomics by LC/MS/MS. (DOCX 16 kb)

Additional file 2: Figure S1. Specific siRNAs down-regulate the expression of LPCAT1. The knockdown of LPCAT1 using two siRNAs in ACHN cells was verified by qRT-PCR (**A**) and western blotting (**B**). LPCAT1 knockdown using two siRNAs in 769P cells was verified by qRT-PCR (**C**) and western blotting (**D**). The results of qRT-PCR are expressed as the means ± SEM of three independent experiments. GAPDH was used as an internal standard. *, *P* < 0.05; **, *P* < 0.01; ***, *P* < 0.001. (TIF 2747 kb)

## Abbreviations

ccRCC: Clear cell renal cell carcinoma
IHC: Immunohistochemistry
LC/MS/MS: Liquid chromatography tandem mass spectrometry
LPC: Lysophosphatidylcholine
LPCAT1: Lysophosphatidylcholine acyltransferase 1
PC: Phosphatidylcholine
PCA: Principal component analysis
PE: Phosphatidylethanolamine
PLS-DA: Partial least-squares-discriminant analysis
SD: Standard deviation
SEM: Standard error of the mean
SM: Sphingomyelin
TMA: Tissue microarray
