# Supplementary material for: Lysophosphatidylcholine acyltransferase 1 upregulation and concomitant phospholipid alterations in clear cell renal cell carcinoma
Source: J Exp Clin Cancer Res. 2017 May 12;36:66. doi: 10.1186/s13046-017-0525-1 (PMC5427523; doi:10.1186/s13046-017-0525-1)
Supplement: Supplementary file 1 — Analysis of tissue and cell lipidomics by LC/MS/MS. (DOCX 16 kb) [file 13046_2017_525_MOESM1_ESM.docx]

**Supplementary**

**Method**

***Analysis of tissue and cell lipidomics by*** ***LC/MS/MS***

Authentic standard compounds were purchased from Avanti Polar Lipids, Inc. (Alabaster, AL, USA), and ammonium acetate was purchased from Sigma-Aldrich Co. (St. Louis, MO, USA). LC/MS grade methanol was purchased from Merck (Darmstadt, Germany), and distilled water (18.02 MΩ) was prepared using a Milli-Q water purification system (Millipore, Billerica, MA).

Accurately weighted tissue (about 100 mg) was homogenized in 1 mL of methanol, and 250 μL of this homogenate was diluted with 750 μL of methanol. Subsequently, 70 μL of the diluted homogenate was mixed with 30 μL of lipid standards (including PC 12:0/13:0, PE 12:0/13:0, So 17:0, Sa 17:0, So-1-P 17:0, Sa-1-P 17:0, SM 12:0, Cer 12:0, GlucCer 12:0, LacCer 12:0, Cer-1-P 12:0 and Cer 25:0) as the internal standard. After vortexing for 1 min, the sample was incubated on ice for 10 min and subsequently centrifuged at 13,200 rpm for 5 min at 4℃. The resulting supernatant (60 μL) was recovered for analysis.

For the cell samples, 3*10^6^ cells were dissolved in 1 mL of chloroform-methanol (3:1, v/v) mixture, ultrasonic homogenized for 1 h and centrifuged at 12,000 rpm for 10 min. Bottom chloroform layer containing lipids was evaporated and redissolved in 90 μL isopropanol-acetonitrile (1:1, v/v) and 10 μL lipid standards (PC 12:0/13:0). After vortexing for 1 min, the sample was incubated on ice for 10 min and subsequently centrifuged at 13,200 rpm for 5 min at 4℃. The resulting supernatant (60 μL) was recovered for analysis.

Thermo Scientific™ Dionex™ UltiMate™ 3000 Rapid Separation LC (Thermo Fisher Scientific, Waltham, MA) system performed ultra-high-performance liquid chromatograph separations with reversed phase C18 column using the gradient conditions shown below. The column was a UPLC HSS T3 column (2.1 mm*100 mm, 1.7 μm, Waters, Milford, MA, USA) operated at 40℃. The mobile phase was 2 mM ammonium formate and 0.1% formic acid in water (A) or methanol (B). The gradient curve was 75% B at 0 min, 100% B at 6 min, 100% B at 20 min, 75% B at 20.1 min, and 75% B at 22.5 min. The flow rate was 0.3 mL/min and injection volume was 1 μL. For the MS, a Thermo Scientific™ Q Exactive™ hybrid quadrupole Orbitrap mass spectrometer equipped with a HESI-II probe was employed (Thermo Fisher Scientific, Waltham, MA). The positive and negative HESI-II spray voltages were 3.7 kV and 3.5kV, respectively, the heated capillary temperature was 320°C, the sheath gas pressure was 30 psi, the auxiliary gas setting was 8 psi, and the heated vaporizer temperature was 320℃. Both the sheath gas and the auxiliary gas were nitrogen. The collision gas was also nitrogen at a pressure of 1.5 mTorr. The parameters of the full mass scan were as follows: a resolution of 70,000, an auto gain control target under 1 × 10^6^, a maximum isolation time of 50 ms, and an m/z range 150–1500. The calibration was customized for the analysis of Q Exactive to keep the mass tolerance of 5 ppm. The calibrated m/zs were 74.09643, 83.06037, 195.08465, 262.63612, 524.26496 and 1022.00341 for positive mode, and 91.00368, 96.96010, 112.98559, 265.14790, 514.28440 and 1080.00999 for negative mode. The parameters of the dd-MS2 scan were as follows: a resolution of 17,500, an auto gain control target under 1 × 10^5^, a maximum isolation time of 50 ms, a loop count of top 10 peaks, an isolation window of m/z 2, a normalized collision energy of 30v and an intensity threshold under 1 × 10^5^. The LC-MS system was controlled using Xcalibur 2.2 SP1.48 software (Thermo Fisher Scientific, Waltham, MA), and data were collected and processed with the same software.

The lipid molecules were annotated by comparing their parent ion and MS/MS ion fragment with those of standard lipid molecules using our own database and the MS prediction tools on the LIPID MAPS public website. The data were normalized based on the tissue weight or cell numbers. The normalized data were further exported to MetaboAnalyst 3.0 for multivariate analysis. Both principal component analysis (PCA) and partial least-squares-discriminant analysis (PLS-DA) were used for modelling the difference between tumour and normal tissues. Paired t-test was used to compare the phospholipid compositions between tumour and normal tissues.
