# Supplementary figures and images for: Lysophosphatidylcholine acyltransferase 1 upregulation and concomitant phospholipid alterations in clear cell renal cell carcinoma
Source: J Exp Clin Cancer Res. 2017 May 12;36:66. doi: 10.1186/s13046-017-0525-1 (PMC5427523; doi:10.1186/s13046-017-0525-1)

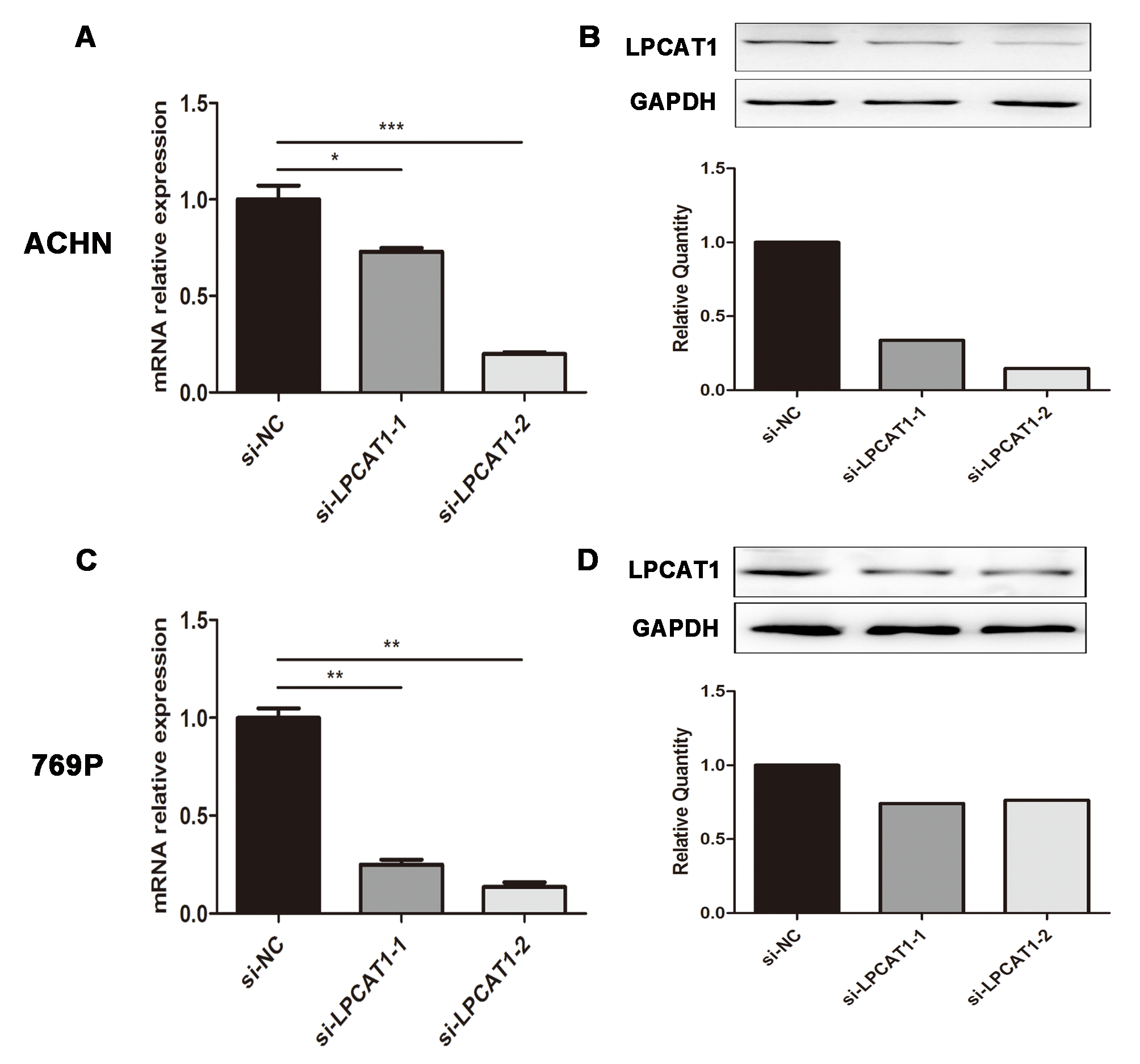

Supplement: Supplementary file 2 — Specific siRNAs down-regulate the expression of LPCAT1. The knockdown of LPCAT1 using two siRNAs in ACHN cells was verified by qRT-PCR (A) and western blotting (B). LPCAT1 knockdown using two siRNAs in 769P cells was verified by qRT-PCR (C) and western blotting (D). The results of qRT-PCR are expressed as the means ± SEM of three independent experiments. GAPDH was used as an internal standard. *, P < 0.05; **, P < 0.01; ***, P < 0.001. (TIF 2747 kb) [file 13046_2017_525_MOESM2_ESM.tif]
